# Industry incentives and antibiotic resistance: an introduction to the antibiotic susceptibility bonus

**DOI:** 10.1038/s41429-020-0300-y

**Published:** 2020-03-19

**Authors:** Chantal M. Morel, Olof Lindahl, Stephan Harbarth, Marlieke E. A. de Kraker, Suzanne Edwards, Aidan Hollis

**Affiliations:** 1 University of Geneva Hospitals and Faculty of Medicine, Rue Gabrielle-Perret-Gentil 4, 1205 Geneva, Switzerland; 2 Global Studies Institute, Rue des Vieux-Grenadiers 10, 1205 Geneva, Switzerland; 3 Department of Business Studies, Uppsala University, Kyrkogårdsgatan 10C Box 513, 751 20 Uppsala, Sweden; 4 Infection Control Programme and Division of Infectious Diseases, University of Geneva Hospitals and Faculty of Medicine, Rue Gabrielle-Perret-Gentil, 41205 Geneva, Switzerland; 5 WHO Collaborating Centre on Patient Safety, University of Geneva Hospitals and Faculty of Medicine, Rue Gabrielle-Perret-Gentil, 41205 Geneva, Switzerland; 6 Department of Health Care Management, Berlin University of Technology, Berlin, Germany; 7 Department of Economics, University of Calgary, 2500 University Dr NW, Calgary, AB T2N1N4 Canada

**Keywords:** Drug development, Antimicrobial therapy

## Abstract

The scarcity of novel antibiotic compounds in a time of increasing resistance rates has begun to ring alarm bells at the highest echelons of government. Large new financial incentives to accelerate antibiotic research and development, such as market entry rewards (MERs), are being considered. However, there is little focus on how to sustain the efficacy of new, promising antibiotics reaching the market. Currently, inappropriate use of antibiotics is commonplace, which has accelerated resistance development. In an attempt to halt this trend, antibiotic stewardship policies are being implemented in many resource-rich settings. Unfortunately, this has not yet had an impact on the amount of antibiotics being prescribed globally. One important hurdle is misalignment of incentives. While governments and health services are incentivized to promote prudent use of this common good, pharmaceutical companies are incentivized to increase volume of sales to maximize profits. This problem must be addressed or else the major efforts going into developing new antibiotics will be in vain. In this paper we outline an approach to realign the incentives of pharmaceutical companies with wider antibiotic conservation efforts by making a staged bonus a component of an MER for antibiotic developers when resistance to their drug remains low over time. This bonus could address the lack of stewardship focus in any innovation-geared incentive.

## Introduction

Antimicrobial resistance (AMR) is attracting political attention because of its present and expected effects on morbidity and mortality as well as financial stress on health systems [1, 2]. Paradoxically, the present focus on antibiotic stewardship to reduce AMR presents also an enormous obstacle to antibiotics development. To safeguard the efficacy of new antibiotics, it is desirable to limit their use to those cases that cannot be successfully treated with existing products. This, however, makes for a terrible business case for investors, since sale volumes for new antibiotics reaching the market remain minimal, at least initially. The low expected return on investment leads, in turn, to reduced interest in antibiotics research and development, with the result that the antibiotic cupboard is increasingly bare. The sad case of Achaogen, which managed to bring a new antibiotic to market, only to immediately fall into bankruptcy owing chiefly to insufficient uptake of its product, shows exactly why many pharmaceutical companies are eschewing investment into antibiotics.

There have been, generally, two types of responses to mitigate this dire situation, which we could describe as “push” and “pull” [3–5]. The push response is for governments to subsidize new antibiotic development, as we see with CARB-X, the Biomedical Advanced Research and Development Authority, and GARD-P. The pull response involves some kind of reward for successfully bringing an antibiotic to market. The UK AMR Review group, in their 2016 report, proposed that it would be important to create a large “market entry reward” (MER) to support new antibiotics, with a reward as high as $1.6 billion [2]. Responding somewhat tentatively to this recommendation, the UK has committed to “develop and test new models for national purchasing arrangements that delink the price paid for antimicrobials from the volumes sold” [6] and there is now much discussion of shaping these into “Netflix”-like payments (also sometimes called insurance or service payments). In the United States, there have been several bills proposed to enhance the profitability of bringing a new antibiotic product to the point of approval. The 2018 REVAMP Act would grant the successful firm a transferable 12-month extension of exclusivity that it could use on another product or sell [7]. This would certainly be worth $1 billion or more if sold to the right bidder. The Infectious Diseases Society of America has expressed support for a well-funded pull incentive [8]. This approach has the strength that it is able to (at least partially) delink the profitability of developing a new antibiotic from the volume sold, which is important as a tool to limit overexploitation of these valuable drugs while also enabling a commercial return on drug development. Determining exactly how to design such a reward is still very much in progress, but a key early study explores how it might be possible to make rewards dependent on specific attributes of any given new antibiotic, with more urgently needed products earning larger rewards [9].

An important aspect of each of these proposals is that they grant the successful company a payment upon approval of a new antibiotic that treats targeted pathogens. Thus, they would increase incentives to develop antibiotics. They would, however, in no cases be dependent on the ongoing effectiveness of the product or the efforts of the company to support stewardship. This point has certainly drawn attention: the European Commission’s 2017 Action Plan on AMR noted that “new economic models need to be developed to incentivise antimicrobial discovery and development *while reconciling these incentives with responsible use* [10].” (our italics). In this paper, we show one way for an MER to be effectively combined with incentives for responsible use. The core idea is the incorporation of an “Antibiotic Susceptibility Bonus” (ASB) into any MER scheme. The ASB would essentially make a part of the MER contingent on the effectiveness of the antibiotic in treating targeted pathogens in the years following market entry.

### Resistance and the role of drug developers

Development of AMR amongst disease-causing pathogens is a biological phenomenon, and as such has a whiff of inevitability. However, this development is amplified and accelerated by ecological pressure from antimicrobials in humans, animals, and the environment. Social and economic behaviour--including over-the-counter sales, inadequate clinical decision making in human medicine, and mass treatment in animal husbandry -- result in overuse of antibiotics and increasing resistance rates among disease-causing pathogens [10, 11]. Recent research suggests that, amongst outpatients, about half of all antibiotics might be unnecessarily prescribed [12, 13].

Despite the importance of the prudent use of antibiotics, overprescription is seen even in countries with an overall restrictive antibiotic prescribing culture [14–16]. Thus far multifaceted interventions combining physician, patient, and public education seem to be the most successful methods to reduce inappropriate prescribing [17]. Other important interventions include enforcement of laws prohibiting over-the-counter sales of antibiotics, and promoting the use of valid point-of-care tests or delayed prescription of antibiotics [18]. Initiatives more recently extended to antibiotic stewardship programs aimed at prescribing physicians [19].

In 2015, the WHO adopted the global action plan to tackle AMR, focusing on five pillars, including optimization of the use of antibiotics [20]. In September 2016, the UN convened a General Assembly in New York to summon strong political commitment in addressing AMR and the development of national action plans. At the time of writing, 58 countries had published their national action plans, which are meant to include strategies to improve appropriate prescribing of antibiotics [21]. Despite all these efforts, the amount of antibiotic prescribing is still increasing in many countries, even in upper-middle-income countries [22], and additional efforts to incentivize their prudent use are needed.

While important steps have been taken by health agencies, the WHO, and other governmental bodies, the pharmaceutical industry has yet to be fully incorporated into this public health effort. It is clear that stewardship is primarily the job of hospitals, doctors, patients, and the agricultural industry. However, there is also a role for pharmaceutical companies that develop and introduce drugs. They determine the indications they will apply to have listed on the antibiotic label. After approval, they choose how to market the product, where, and to whom. These choices are material to the evolution and spread of resistance. Pharmaceutical companies have a role to play and the approach of the ASB is to ensure that their financial motivations are well aligned with the social goals of getting the highest benefit from new antibiotics.

**Fig. 1:**
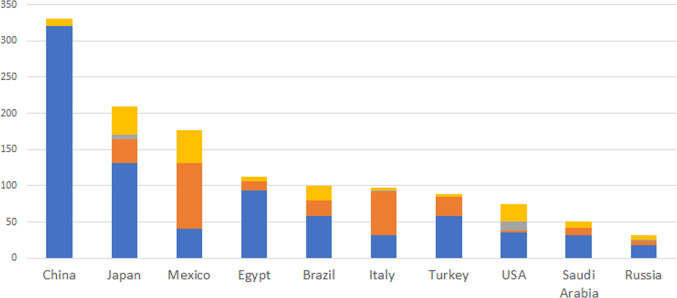
2017 spending on antibiotic promotion by type of promotional activity (US$ million). Source: IQVIA ChannelDynamics® database.  Salesforce to specialists,  Salesforce to primary care physicians,  Salesforce to nurses, pharmacists,  Conferences, KOLs, brochures, TV

While there has been a significant reduction in new antibiotics arriving on the market, there continues to be substantial investment in the promotion of antibiotics, suggesting that industry has a real potential role in determining how these products are used. Using 2017 global data from IQVIA ChannelDynamics®, we calculated the average spending on promotion as a fraction of total revenues across all drugs (including generics). Excluding antibiotics (ATC code J1), the average promotional spend was 5.7% of revenues, while antibiotics were somewhat lower at 4.2%, but still substantial. Moreover, much of this spending is in markets in middle-income countries, where AMR is rampant. Figure 1 shows the type and location of spending in leading markets.

It is helpful to understand what promotion is aimed at. In 2017, the leading molecule for promotional spending, according to IQVIA, was amoxicillin–clavulanic acid (9% of total antibiotic promotion); and by far the largest share of spending on this product (25%) was targeted at promoting Augmentin®, the branded equivalent, although amoxicillin–clavulanic acid has been generic in the United States and most other countries since 2002. In effect, pharmaceutical innovators have substantial capacity to influence which products and how much of them are used; if not, they would not invest so heavily in promotional activities.

### The antibiotic susceptibility bonus

The ASB is intended to function as the “performance” component of an MER,^1^which would be conditional on pathogen susceptibility to the product (the product’s ability to stave off resistance) at, for example, 5, 10, 15, and 20 years following market approval. This would require governments that supported an MER to commit to making conditional payments in the future, subject to agreed performance measures, to the patentee or assignee of a novel, qualifying antibiotic. Just to help make this more concrete, instead of offering a $1.6 billion MER, we propose, for example, a $1 billion MER, with conditional payments of up to $400 million at years 5, 10, 15, and 20, if the product meets prespecified susceptibility targets at those dates. Accounting for a 10% cost of capital, such payments would have about the same value as $1.6 billion, if the product met the targets. If it did not meet those targets, of course, the total payment to the firm would be less.

Adding the ASB component into an MER would offer three benefits. First, the ASB would help counter the incentive to prioritize sales over longer-term efficacy and, as such, would directly align public health and commercial goals. It would give the patentee a financial motivation to consider stewardship of its product. As resistance is generally related to utilization of the antibiotic, the ASB would incentivize companies to reduce inappropriate sales, to reserve the product for treatment of serious infections caused by pan-resistant bacteria, and to promote the product in line with those goals. The bonus would then make up for the income foregone in limiting sales. While pharmaceutical companies generally express a desire to participate in stewardship of their products, such participation is always more likely to be effective when there is a financial motivation.

Second, the ASB would help to guide firms to advance candidate products that show the greatest likelihood of long-lasting effectiveness. While of course no firm wants to bring to market a product against which bacteria will quickly develop resistance, the ASB would emphasize early exploration resistance properties and methods for their prediction and detection.

Third, the ASB would help to protect the MER mechanism. It is certain that the MER would come under intense criticism if, following the payment of say $1.6 billion for approval of a new antibiotic, the product were to become obsolete over a short time period due to resistance. It would then be extremely difficult to sustain support for future MERs. Making a substantial fraction of the MER payable over time, upon the requisite showing of ongoing susceptibility of the product, would help mitigate this risk.

Against these benefits, the ASB would create two types of costs. First, the conditionality of the ASB on performance of the MER would reduce the overall incentive to invest in antibiotic research, since firms would know that there was a possibility that their product might not meet the performance standards of the ASB. This effect could be mitigated by increasing the total size of the MER, including the ASB. This would, however, create some uncertainty about the total budgetary requirement. Moreover, it is common to claim that pharmaceutical companies need the payment immediately to address cash-flow requirements. This problem could be mitigated by the company selling a fraction of its right to the ASB payments, for example, to a royalty trust.

Second, assessing the performance of the MER would involve some costs and would undoubtedly raise controversies. However, we believe that the costs are relatively small and would yield much value in any case. We discuss how to best minimize controversies below.

### How the ASB would affect behaviour

Pharmaceutical companies are not the primary drivers of antibiotic stewardship. Once a product has been sold to wholesalers, pharmacies, or hospitals, the pharmaceutical company no longer has control over how it is used. However, there are several ways that the company can influence the potential development of AMR, starting with developing a product that is not easily subject to resistance. At present, companies have little incentive to protect their products from resistance: with expected exclusivity of about 12 years, firms know that their money-earning opportunities are limited. To maximize profits during those 12 years, firms may do things to increase profits in ways that jeopardize the value of the product to society over time:

- Firms may attempt to ensure that their product has many indications, even for infections that are adequately treated by existing products. For example, a company conducted numerous clinical trials evaluating the use of telithromycin for acute otitis media and pharyngitis, infections that are relatively well treated by other antibiotics [23].
- Firms might promote their product extensively, leading to substitution for other existing products or inappropriate use. While there is increasing awareness of the importance of responsible promotion, heavy promotion in some markets continues. Even in the United States, as recently as 2010, there was enforcement regarding the off-label promotion of an agent indicated for use in cystic fibrosis [24].
- Price their product to increase sales and
- encourage or facilitate inappropriate use in companion animals or livestock [25].

These are not meant to be accusations; generally we observe firms attempting to be responsible with the sale of new antibiotics, but the underlying incentives guide firms toward profit maximization. The ASB would provide an economic incentive for firms to care about the impact of their choices on resistance.

In aiming for the bonus, companies would be incentivized to support human use only, antibiotic stewardship, infection prevention and control measures, and relevant environmental policies such as those surrounding careful disposal of postproduction effluent. Indeed, it would be directly in the company’s interest to minimize the potential emergence and transmission of resistance to its novel compound that could at some point affect its clinical effectiveness either directly or through cross-resistance. In effect, a company pursuing this bonus would be incentivized to protect the clinical effectiveness of all antibiotics with a similar mechanism of action.

### How the scheme fits within the broader antibiotic incentives debate

So-called “fully delinked” MERs, buyouts, and service provision models propose to delink company remuneration from unit sales through large one-off or staged rewards for bringing a product to market [9, 26, 27]. Alternatively, in the “partially delinked” MER model, a large-scale reward would be given but leave in place the ability to sell units normally onto the market after approval [4, 5, 28]. The ASB could be combined with either fully or partially delinked models or even operate on a stand-alone basis. In any case, the ASB would help to align firms’ incentives with social goals of preserving antibiotic effectiveness.

The idea of linking the reward scheme for antibiotics to their effectiveness has been raised within the incentives debate before. For example, Kesselheim and Outterson proposed that the US-Food and Drug Administration (FDA) could extend patent exclusivity on antibiotics as a reward for continuing effectiveness of the molecule [29]. In a follow-up publication these authors proposed the use of financial rewards, but the practical details of how to implement such a scheme were not worked out [30]. Outterson et al. proposed an “Antibiotic Health Impact Fund” that would include specific payments geared to meeting conservation targets [31]. But again this idea was not fully developed. In particular, prior research has not yet considered how conservation would be assessed, an important part of the ASB functioning.

### Key aspects of the ASB

From the perspective of theory, the ASB makes sense. But there are still key questions about bonus magnitude, measuring susceptibility in an accurate and representative manner, safeguarding clinical access, ensuring that only deserving products (those that are safe and truly useful) are rewarded, and how to deal with ensuring conservation after patent expiry.

#### Baseline magnitude of the bonus (prior to weighting)

The size of the bonus will need to be determined on a product-by-product basis, taking into account both epidemiological and market considerations. As with any reward determined extra market, the calculation of an optimum bonus can be done in several ways. The bonus must be high enough to motivate a meaningful response from industry to aim for it, while respecting taxpayers or other funders of the scheme. To incentivize industry, the expected bonus size must exceed the loss in profits from constraining use, the latter of which depends on the epidemiological patterns, the regulatory environment, and the market.

The size of the ASB rewards should be based on the concept that the ASB payments must be large enough to discourage the innovator from promoting uses of the product that would have a relatively low sales value and a high cost in terms of increasing resistance. For example, a use with relatively low sales value could be in a case where the firm seeks to sell for an indication with many competing products. A use with high cost in terms of increasing resistance could, for example, be heavy sales to a hospital with a record of weak stewardship. In effect, the ASB is intended to provide a financial incentive for the innovator to become more attentive to whether sales are likely to increase resistance. The larger the size of the ASB, the stronger this incentive would be.

One concern is that if the ASB were large enough to shift behavior significantly, it might also represent an undeserved windfall to innovative firms. We do not believe that this concern is warranted. One of the significant failings of our current system of incentives for antibiotics is that the patentee is rewarded only based on the first decade of sales, regardless of whether the product continues to be effective after generic entry. With the ASB, an antibiotic with enduring effectiveness could be rewarded more generously than one which suffered a rapid decline in usefulness because of resistance. Thus, the ASB would not only optimize incentives for utilization, but also for investment in developing new, more promising antibiotics.

#### Susceptibility

Susceptibility is at the heart of this conservation-geared ASB. However, susceptibility to novel antibiotics is not currently measured on a routine, large-scale basis. Indeed, thus far, epidemiological surveillance of AMR has been limited in many regions of the world and focussed on few infections, few pathogens, and a limited number of antibiotics, which have been used for decades. This is in large part due to a lack of easily accessible, high quality, harmonized data; differences in sampling indications, methods and rates, differences in microbiologic methods between settings, and data validity issues in many low-resource settings. Standardized surveillance has largely been limited to high-resource settings reporting on AMR in bloodstream infections. However, bloodstream infections represent just the “tip of the iceberg” and will not provide early warning signs of emerging resistance. Moreover, most surveillance systems have a long delay in reporting of more than a year. Regulators also do not routinely require resistance surveillance from companies once a new drug reaches the market. Since 2007 the FDA Amendments Act requires companies to complete postmarketing approval studies, which can, but do not need to, include research into drug resistance through in vitro laboratory safety studies [32]. In Europe, postmarketing surveillance studies on emergence of AMR are mentioned in the European Medicines Agency’s risk management plan, but no clear requirements or repercussions are formulated [33]. Some antibiotics developers are nonetheless collecting their own AMR data (e.g. Pfizer’s Atlas program: https://atlas-surveillance.com). However, the objectives of such initiatives are different from those for public health purposes, or the ASB, and sampling schemes are often unclear and external validity is limited. For example, their current sampling frame would not provide a representative picture of possible AMR emergence in a specific region. Moreover, given the conflicts of interest caused by the relationship between resistance detection and product sales, the resulting data may be of limited value. Overall, given the current lack of suitable resistance surveillance, ASB surveillance will likely need to follow a more targeted approach and be conducted by public health authorities parallel to currently established procedures—at least until broader surveillance is better established, possibly with tightly steered collaboration involving both public and private actors. Although the ASB would require some investment in surveillance, this would have value beyond the mechanism itself.

In theory, ASB surveillance could use average measures of the minimum inhibitory concentration to determine, accepted (e.g. by EUCAST) clinical breakpoints (susceptible/intermediate/resistant or SIR), and a level of acceptable resistance to determine eligibility. However, breakpoints can change over time, and offering a reward would significantly increase pressure from companies to alter them. Deviation from the epidemiological cutoff values, the ECOFF in Europe (similar to the ECV used in the United States) could have substantial advantages over the use of breakpoints in the context of the ASB. ECOFFs are calculated from a vast number of isolates of a particular bacterial species from diverse sources globally, resulting in a known population distribution. They allow us to determine when detected resistance has been acquired since the drug was launched or rather was inherent, deriving from the wild-type distribution of the bacterium for a particular drug. While ECOFFs have little clinical relevance compared with breakpoints, crucially they remain constant over time and relatively consistent across systems (e.g. EUCAST, CLSI, FDA).

#### Access to novel antibiotics

There is potential for the effects of the ASB to be too strong and possible problems need to be addressed from the start through careful design of the mechanism. In particular, if the magnitude of the bonus is sufficiently high compared with expected sales, there is a risk that this would create a disincentive to sell in any country, below a clinically optimal point. In order to ensure that the product is supplied where and when it is needed, passive supply chain arrangements will be required. For example, drug companies seeking the bonus would agree to provide their product when requested by qualifying buyers who met requirements for responsible use (including pooling purchasers with some downstream conditions). Supply would thus be triggered by justified requests for the product rather than by promotional activities of the companies. The formality of stating demand in this way lowers the chances of superfluous use and improves supply where there is currently unmet demand in less privileged markets. Such arrangements are likely to be appropriate while use of the novel antibiotic in question remains highly restricted, but if the need for the product were to increase the scheme would have to utilize alternative logistic arrangements. While the ASB is intended to help conserve precious new antibiotics it is imperative that it not impede justified clinical access.

#### Utility of the antibiotic

The ASB should be granted only to novel products that are truly useful for prescribers to treat patients with multidrug-resistant infections. However, randomized controlled trials (RCTs) required for market approval will not necessarily provide evidence of the clinical efficacy in each specific patient group. Due to the limited number of patients, and the complexities of recruiting these patients for RCTs [34], most trials will include a mix of patients with drug-susceptible and resistant infections and will assess noninferiority. Therefore, the usefulness and effectiveness of the product in real-world settings needs to be determined postlaunch. Using unit sales as an indicator of usefulness (unit sales being the traditional indicator of usefulness in a normal market) would incentivize sales and increase the risk of driving resistance as it would reinstate the perverse incentive that this mechanism seeks to remove. Therefore, other indicators of usefulness need to be explored in designing the ASB, including their estimated overall social value, in order to weight the bonus. The proposal by Rex and Outterson provides a good basis for this [9].

#### Weighted bonus magnitude

When ASB rewards are ultimately determined (e.g. at the 5, 10, 15, and 20-year marks), the baseline magnitude is modified according to the performance of the drug and of the company. More specifically, magnitude is weighted by access (e.g., proportion of justified product requests that are met by the company, binary qualifier using a minimum access threshold) and susceptibility (e.g., proportion of isolates with minimum inhibitory concentrations deviating from the ECOFF to an acceptable extent). Both access and susceptibility must utilize a floor threshold—a minimum below which eligibility for the ASB is rejected—in order to hedge against high levels of risk taking (maximizing unit sales). It is important for the rules of the ASB to be clear so that companies can form realistic expectations about the value of ASB payments.

#### Postpatent years

How would the ASB operate once generic entry occurs? On average, new drugs enjoy about 12 years of exclusivity before generic entry, at which point prices drop. The ASB does not directly affect generic drugs, but is instead designed to affect how antibiotics are introduced to the market in the first 10–12 years. Generic manufacturers normally do not engage in promoting products to physicians or hospitals and do not undertake market-expanding research. As such, there is no reason to assume that generic entry would hugely increase sales volumes. The fact that the ASB would not be able to influence behaviour of generic manufacturers will therefore not be as important. The ASB is targeted at the crucial years before generic entry, when the innovator firm’s choices, including promotion of use, will affect how the antibiotic comes into use. Offering the ASB payments in the postpatent period (e.g. at 15 and 20 years postlaunch) provides incentives to the innovator to market the product appropriately before generic entry so that the patterns of use that follow, even once generic competitors enter, support conservation of the antibiotic.

## Conclusion

Without deliberate efforts to include all key stakeholders in our fight for prudent use of novel antibiotics, the efficacy of any new antibiotics reaching the market could be quickly eroded, and an MER subverted. An ASB would direct industry efforts toward more targeted, appropriate use that protects long-term product efficacy and avoids profit-driven product promotion as it reduces the dependence on high-volume sales. Such market intervention is well aligned with the public health goal of encouraging development of antimicrobials with a more durable profile over the years to come. There is an urgent need for further research into how to incorporate pharmaceutical companies in the effort to preserve antibiotic effectiveness.

## Footnotes

1 The ASB can actually be applied to any partially or fully delinked reward mechanism.
